# The 5HT_1a_ Receptor Agonist 8-Oh DPAT Induces Protection from Lipofuscin Accumulation and Oxidative Stress in the Retinal Pigment Epithelium

**DOI:** 10.1371/journal.pone.0034468

**Published:** 2012-04-03

**Authors:** Prajitha Thampi, Haripriya Vittal Rao, Sayak K. Mitter, Jun Cai, Haoyu Mao, Hong Li, Soojung Seo, Xiaoping Qi, Alfred S. Lewin, Carl Romano, Michael E. Boulton

**Affiliations:** 1 Department of Anatomy and Cell Biology, University of Florida, Gainesville, Florida, United States of America; 2 Department of Molecular Genetics & Microbiology, University of Florida, Gainesville, Florida, United States of America; 3 Retina Research, Alcon Laboratories, Inc, Fort Worth, Texas, United States of America; Duke University, United States of America

## Abstract

Age-related macular degeneration (AMD), a major cause of blindness in the elderly, is associated with oxidative stress, lipofuscin accumulation and retinal degeneration. The aim of this study was to determine if a 5-HT_1A_ receptor agonist can reduce lipofuscin accumulation, reduce oxidative damage and prevent retinal cell loss both *in vitro* and *in vivo*. Autophagy-derived and photoreceptor outer segment (POS)-derived lipofuscin formation was assessed using FACS analysis and confocal microscopy in cultured retinal pigment epithelial (RPE) cells in the presence or absence of the 5-HT_1A_ receptor agonist, 8-OH DPAT. 8-OH DPAT treatment resulted in a dose-dependent reduction in both autophagy- and POS-derived lipofuscin compared to control. Reduction in autophagy-induced lipofuscin was sustained for 4 weeks following removal of the drug. The ability of 8-OH DPAT to reduce oxidative damage following exposure to 200 µM H_2_O_2_ was assessed. 8-OH DPAT reduced superoxide generation and increased mitochondrial superoxide dismutase (MnSOD) levels and the ratio of reduced glutathione to the oxidized form of glutathione in H_2_O_2_-treated cells compared to controls and protected against H_2_O_2_-initiated lipid peroxidation, nitrotyrosine levels and mitochondrial damage. SOD2 knockdown mice, which have an AMD-like phenotype, received daily subcutaneous injections of either saline, 0.5 or 5.0 mg/kg 8-OH DPAT and were evaluated at monthly intervals. Systemic administration of 8-OH DPAT improved the electroretinogram response in SOD2 knockdown eyes of mice compared to knockdown eyes receiving vehicle control. There was a significant increase in the ONL thickness in mice treated with 8-OH DPAT at 4 months past the time of MnSOD knockdown compared to untreated controls together with a 60% reduction in RPE lipofuscin. The data indicate that 5-HT_1A_ agonists can reduce lipofuscin accumulation and protect the retina from oxidative damage and mitochondrial dysfunction. 5-HT_1A_ receptor agonists may have potential as therapeutic agents in the treatment of retinal degenerative disease.

## Introduction

Age-related macular degeneration (AMD) is the major cause of blindness in the elderly in developed countries, with more than 1.7 million Americans over age 65 having visual impairment due to AMD [1]. Although AMD has a complex etiology which is determined by a variety of environmental factors and inherent genetic susceptibilities, there is a consensus that the retinal pigment epithelium (RPE) plays a central role in its pathogenesis. Elevated levels of the age pigment lipofuscin and oxidative stress are associated with a variety of retinal degenerations including AMD, and there is considerable circumstantial evidence linking lipofuscin with the pathobiology of AMD [2], [3], [4], [5], [6]. The highest density of lipofuscin is located in the central retina where the density of rod outer segments is at its highest and where there is a preferential loss of rod cells in AMD [7]. Cumulative oxidative damage, chronic phototoxicity, lysosomotropic damage and occupation of cytoplasmic volume in RPE cells by lipofuscin throughout life will contribute to RPE dysfunction and put added stress on a highly metabolically active cell type [8], [9]. Longitudinal monitoring of fundus autofluorescence demonstrates that the presence of local areas or “hot spots” of lipofuscin in the RPE layer are a risk for progression of AMD [3], [4], [10], [11] and these areas exhibit a variable loss of retinal sensitivity [12]. Lipofuscin levels are also elevated in rodent models for AMD [13], [14], [15]. Lipofuscin can be observed in early drusen and these granules may serve as an immunogenic stimulus for the localized activation of dendritic cells, complement activation and the deposition of inflammatory factors [16], [17]. Given the increasing evidence for lipofuscin involvement in AMD there is a growing interest in the development of therapeutic agents to both prevent lipofuscin formation and eliminate preexisting lipofuscin. These studies have primarily focused on slowing the retinoid cycle and reducing the accumulation of retinoid derivatives such as A2E in lipofuscin [6], [18], [19]. However, while offering some success, these approaches have been associated with slowed dark adaptation and reduced ERG responses [19], [20].

The 5-HT_1A_ receptor is a subtype of the G protein-coupled 5-HT receptor family that binds the endogenous neurotransmitter serotonin (5-hydroxytryptamine, 5-HT) [21]. The 5-HT_1A_ receptor is the most widespread of all the 5-HT receptors and is expressed in both the neural retina and the RPE [22], [23], [24]. 5-HT_1A_ agonists provide protection against a variety of insults, including: serum deprivation [25], staurosporine [26], anoxia [27], excitotoxicity [28] and oxidative damage [29]. Furthermore, we have recently reported that 5-HT_1A_ agonists can provide potent and complete functional and structural protection from severe blue-light induced photooxidative damage [22], [23]. ERGs were significantly higher in light exposed rats treated with 5-HT_1A_ agonists and retinas were devoid of histological lesions compared to vehicle only controls. In addition, 5-HT_1A_ agonist caused a decrease in microglial activation and recruitment as well as reduced complement deposition in the outer retina.

In this study we report that the 5-HT_1A_ agonist, 8-hydroxy-2-(di-*n*-propylamino)-tetralin (8-OH DPAT), is able to reduce the accumulation of both autophagic-derived and photoreceptor outer segment-derived lipofuscin, increase antioxidant protection and reduce oxidative damage in cultured human RPE cells as well as being able to reduce lipofuscin accumulation and improve visual function in an animal model of AMD.

## Results

### 5-HT_1A_ agonist reduces lipofuscin accumulation in cultured RPE cells

**Figure 1:**
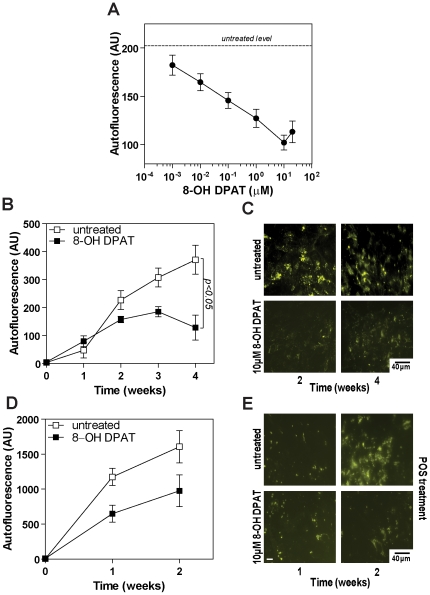
8-OH DPAT reduces lipofuscin accumulation in cultured RPE cells. **A** - The effect of different concentrations of 8-OH DPAT on lipofuscin accumulation from autophagy in human ARPE19 cells. Cells were maintained in basal medium and received 10 µM 8-OH DPAT every two days for four weeks. **B** - Autofluorescent intensity of ARPE19 cells treated with 10 µM 8-OH DPAT every two days for up to 4 weeks. Control cells received vehicle alone. **C** - Fluorescent micrographs of ARPE19 cells as described in B showing reduced lipofuscin granules in 8-OH DPAT treated cells compared to controls. **D** - Autofluorescent intensity of ARPE19 cells fed POS treated with 10 µM 8-OH DPAT every two days for up to 14 days. Control cells received POS and saline vehicle alone. **E** - Fluorescent micrographs of ARPE19 cells described in D showing reduced lipofuscin granule accumulation in 8-OH DPAT treated cells fed POS compared to untreated controls. Fluorescence intensity was determined by flow cytometric analysis. Data represent the mean of three experiments. Bar marker is 50 µM.

8-OH DPAT treatment resulted in a dose-dependent reduction in both autophagy- and POS-derived lipofuscin accumulation (Fig. 1). The cells showed no apparent morphological change apart from the reduction of lipofuscin. Autophagy-derived lipofuscin accumulation was reduced by 8-OH DPAT treatment over a 4 week period compared to untreated control (p<0.01) with approximately 50% reduction in autofluorescence at 10 and 20 µM 8-OH DPAT (Fig. 1a). Based on these observations, we elected to use 10 µM 8-OH DPAT for the majority of our subsequent experiments. We next assessed the effect of 10 µM 8-OH DPAT on the time-dependent accumulation of autophagy-derived lipofuscin over a 4 week period. We observed a significant decrease of 30% and 67% (p<0.01) after 3 and 4 weeks treatment respectively compared to untreated control (Fig. 1b) and that the beneficial effect of 8-OH DPAT became maximal from 3 weeks onward. The 8-OH DPAT induced decrease in lipofuscin accumulation could be visually verified using fluorescence microscopy (Fig. 1c).

8-OH DPAT was also able to reduce lipofuscin accumulation derived from phagocytosed photoreceptor outer segments (POS) by 28% over 14 days compared to untreated control (p<0.05) (Fig. 1d) and this reduction was visually confirmed by fluorescence microscopy (Fig. 1e). There was no significant difference between the two concentrations (1 and 10 µM) of 8-OH DPAT tested. The magnitude of autofluorescence caused by phagocytosis of POS is much greater than that caused by autophagy. (Note the scale differences in the ordinates of Fig. 1a and 1d.) Consequently, the decrease in phagocytic lipofuscin caused by 8-OH DPAT must be largely independent of its inhibition of autophagy.

**Figure 2:**
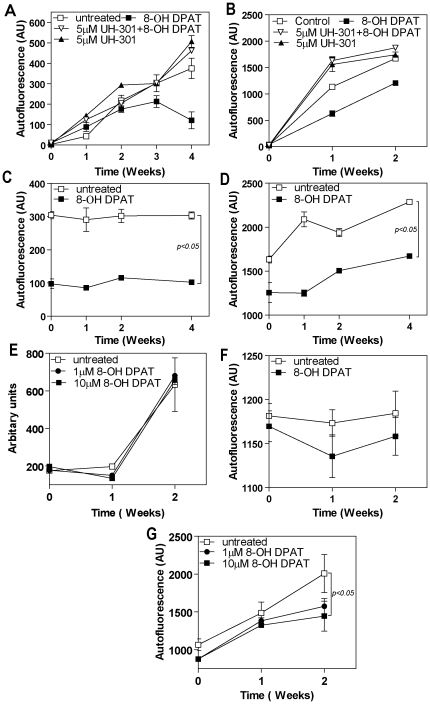
The effect of 8-OH DPAT is sustained following discontinuation but 8-OH DPAT has only a minimal effect on pregenerated lipofuscin. **A, B** - The effect of the selective 5-HT_1A_ receptor antagonist, UH-301 (5 µM) on 8-OH DPAT (10 uM) reduction in autophagy- (**A**) and POS-derived (**B**) lipofuscin accumulation in cultured RPE. **C, D** - To determine if the effect of 8-OH DPAT was sustained following discontinuation of 8-OH DPAT treatment, 8OH DPAT treatment was discontinued after 28 days and the cells maintained in basal medium (**C**) or fed POS (**D**) for a further 28 days. **E, F, G** - To assess the ability of 8-OH DPAT (10 uM) to remove pregenerated lipofuscin, 8-OH DPAT was added every second day for 28 days after: **E** – 28 days accumulation of autophagy-derived lipofuscin; **F** - cultures were fed mature lipofuscin granules; **G** - 14 days accumulation of POS-derived lipofuscin. Data represent the mean of three experiments.

To confirm that 8-OH DPAT was reducing lipofuscin by binding the 5HT_1A_ receptor we assessed the effect of the 5HT_1A_ receptor antagonist UH-301. UH-301 blocked the 8-OH DPAT-induced reduction in both autophagy- and POS-derived lipofuscin accumulation in cultured RPE (Fig. 2a,b).

To test whether reduction in lipofuscin associated with 8-OH DPAT would persist after the drug was removed, we measured autofluorescence for an additional 4 weeks after cessation of treatment. For autophagy-induced lipofuscin, this was, in fact, the case: reduction in autofluorescence was maintained for 28 days after treatment stopped (Fig. 2c). In the case of POS-derived lipofuscin, although autofluorescence accumulation remained absent for the first week after discontinuation of 8-OH DPAT, the rate of lipofuscin accumulation eventually returned to that seen in control cultures fed POS but not receiving 8-OH DPAT treatment (Fig. 2d). However, the overall decrease in lipofuscin accumulation prior to discontinuation of 8-OH DPAT was maintained. Having shown that 8-OH DPAT was able to inhibit lipofuscinogenesis we next wished to determine if 8-OH DPAT could reduce levels of prexisting lipofuscin. 8-OH DPAT did not lead to significant reduction in preformed lipofuscin that was derived from autophagy nor did it reduce autofluorescence in ARPE19 cells fed mature human lipofuscin granules (Fig. 2e,f). However, there was a small, but significant (p<0.05), reduction in pre-existing POS-derived lipofuscin (Fig. 2g).

### 5-HT_1A_ agonist reduced oxidative damage in cultured RPE cells

**Figure 3:**
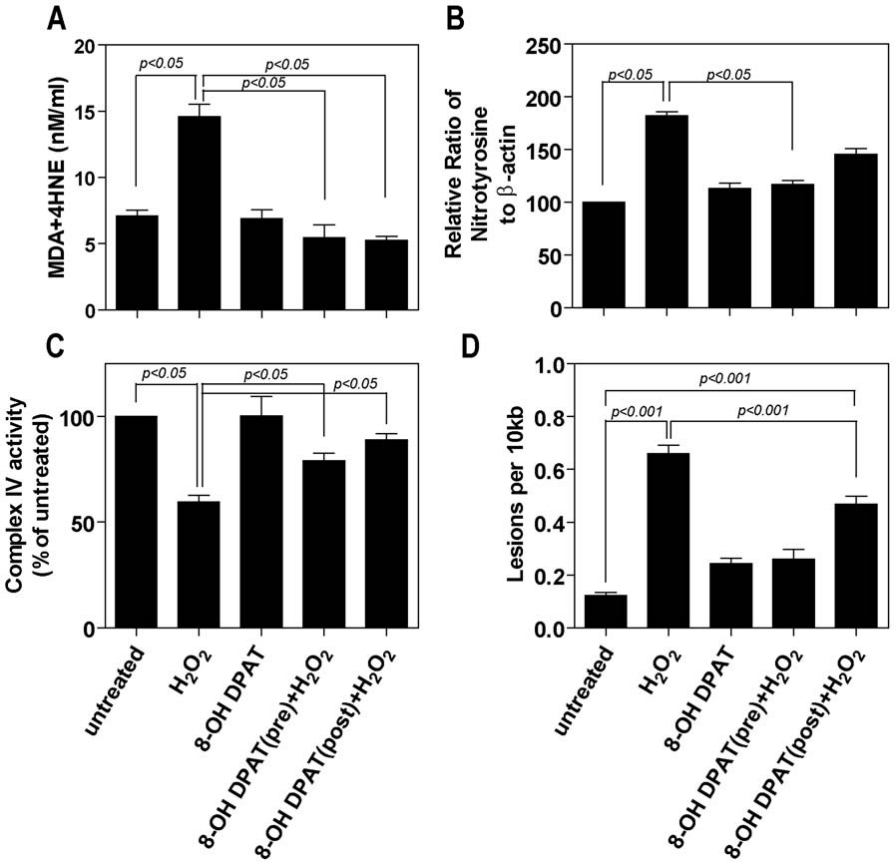
8-OH DPAT reduces lipid peroxidation and peroxynitrite accumulation and protects against mitochondrial damage in cultured RPE cells exposed to H_2_O_2_. Cells were exposed to H_2_O_2_ (200 µM) for 1 hour and either pre-or post treated with 8-OH DPAT (10 µM) for 24 hours. In the case of pretreatment all measurements were made 24 hr after H_2_O_2_ and for post treatment 8-OH DPAT was added immediately following H_2_O_2_ exposure. **A** - The level of lipid peroxides (4HNE/MDA) was determined using a commercially available kit from OxisResearch. **B** – Nitrotyrosine levels were assessed by Western blotting using a polyclonal antibody and band intensities were normalized against β-actin. **C** - Cytochome *c* oxidase levels were determined using a Complex IV ELISA assay. **D** - Mitochondrial DNA damage was analyzed as previously described using the long chain PCR reaction. Data represent the mean of three experiments.

H_2_O_2_ treatment led to a 65% increase in lipid peroxidation as measured by levels of 4HNE and MDA, and to a 70% increase in the level of nitrotyrosine-modified proteins. These values were significantly reduced to control levels in cells treated with 8-OH DPAT for 24 hours (Fig. 3a,b). There was no significant difference whether 8-OH DPAT was applied pre- or post-exposure to H_2_O_2_.

### 5-HT_1A_ agonist protects against mitochondrial damage in cultured RPE cells

Since superoxide radicals are largely derived from mitochondria in the RPE we assessed if 8-OH DPAT could protect against mitochondrial damage. 8-OH DPAT alone had no effect on Complex IV activity. Pre-treatment of ARPE19 cells with 8-OH DPAT for both 3 (data not shown) and 24 hours reduced the impact of oxidative stress on the level of cytochrome *c* oxidase (Complex IV) by up to 50% (Fig. 3c). Post-treatment with 8-OH DPAT for 3 hours (data not shown) had no effect while 24 hour post-treatment did show a significant improvement of around 65%. Furthermore, both pre and post-treatment with 8-OH DPAT significantly protected cultured RPE cells from H_2_O_2_-induced mitochondrial DNA damage and reduced the number of lesions per 10 kb by greater than 50% (Fig. 3d).

### 5-HT_1A_ agonist reduces superoxide anion generation and increases antioxidant capacity in cultured RPE cells

**Figure 4:**
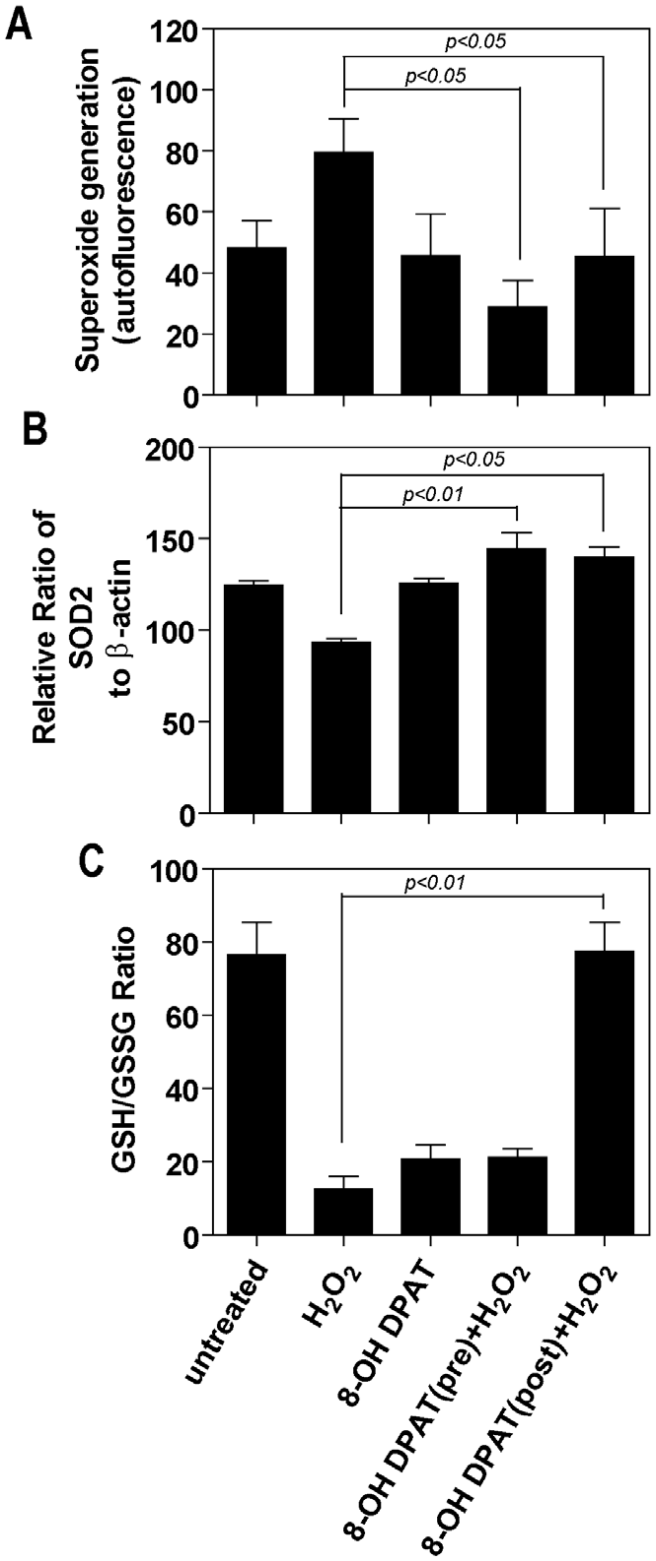
8-OH DPAT reduces superoxide anion generation and increases antioxidant capacity in cultured RPE cells. Cells were exposed to H_2_O_2_ (200 µM) for 1 hour and either pre-or post treated with 8-OH DPAT (10 µM) for 24 hours. In the case of pretreatment all measurements were made 24 hr after H_2_O_2_ and for post treatment 8-OH DPAT was added immediately following H_2_O_2_ exposure. **A** - Superoxide generation was measured using FACS analysis following staining with MitoSOX and results are expressed as the mean fluorescence intensity. **B** - SOD2 levels were determined by Western blot analysis. **C** - The ratio of reduced glutathione (GSH) to the oxidized form of glutathione (GSSG) was measured by ELISA. Data represent the mean of three experiments.

Treatment with H_2_O_2_ stimulated a 102% increase in the generation of superoxide anions (Fig. 4a). 8-OH DPAT was able to reduce oxidative stressor-induced superoxide generation when given either before or after H_2_O_2_ treatment. However, pre-treatment appeared to be the most effective. A 3 and 24 hour 8-OH DPAT pre-treatment of H_2_O_2_-exposed cells resulted in a greater than 67% and 35%, respectively, reduction in superoxide anions. Post-treatment was significantly less effective compared to pre-treatment, and a significant reduction in superoxide anions was only observed for cells exposed to H_2_O_2_ and then treated with 8-OH DPAT for 24 hours (Fig. 4a). There was no significant difference between 1 or 10 µM 8-OH DPAT on the reduction in superoxide generation. Interestingly, although 8-OH DPAT alone had no significant effect on endogenous superoxide anion generation in the absence of oxidative stressor, in the presence of H_2_O_2_ it was able to reduce levels of superoxide anions to significantly less than in untreated control cultures. This effect may be due to the oxidative stressor activating one or more of the antioxidant pathways.

Treatment with 8-OH DPAT led to a 42% increase in MnSOD following H_2_O_2_ exposure compared with oxidatively stressed cells not receiving 8-OH DPAT (Fig. 4b). The increase in MnSOD levels were similar whether 8-OH DPAT was given before or after H_2_O_2_ treatment. Cells treated with both H_2_O_2_ and 8-OH DPAT showed a significant decrease in the GSH/GSSG ratio indicating an increase in reduced glutathione compared to untreated controls (Fig. 4c). Exposure to 8-OH DPAT for 24 hours post H_2_O_2_ resulted in an increase in the GSH/GSSG ratio to a level seen in untreated cells. By contrast, preexposure to 8-OH DPAT only demonstrated a small increase in the GSH/GSSG ratio compared to cells treated with H_2_O_2_ alone (Fig. 4c).

### 5-HT_1A_ agonist mediates neuroprotection in a mouse model of AMD

**Figure 5:**
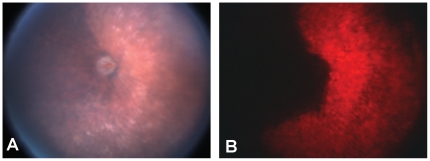
Extent of retinal transduction with AAV-VMD2-*SOD2* Rz. Digital fundus images were made 4 months following subretinal injection of the AAV-VMD2-*SOD2* Rz, which also contained the mCherry gene as a marker of genetic transduction. As previously reported [15] we routinely observe 60–80% transduction of the RPE. This was revealed as red-fluorescence using a long wave length filter (A) and as thinning of the retina (hypopigmentation) as a consequence of knockdown of MnSOD (B).

**Figure 6:**
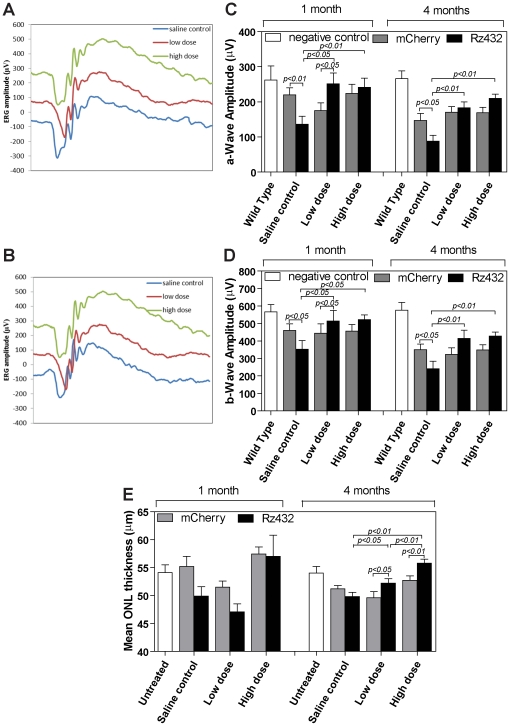
ERG a-wave and b-wave amplitudes and retinal thickness measured in AAV-ribozyme SOD2 knockdown eyes and control eyes treated with 8-OH DPAT. Eyes received injection of AAV-*SOD2* ribozyme or AAV-mCherry and animals received subcutaneous 8-OH DPAT or saline control for up to 4 months. ERGs were obtained at 1 and 4 months following virus injection: **A & B** - ERG wave-forms from mice treated with AAV-mCherry (**A**) or AAV-VMD2-*SOD2* Rz (**B**). Simultaneous full-field ERG measurements were recorded in dark-adapted mice four months after injection with AAV. The scale units on the ordinate are 100 microvolts. The graphs show wave forms for mice treated with saline, blue lines, low dose (0.5 mg/kg) 8-HO-DPAT, red lines, or high dose (5 mg/kg) 8-HO-DPAT, green lines. **C** – a-wave; **D** – b-wave. **E** - ONL thickness measured by SD-OCT at 1 and 4 months. Untreated wild type animals acted as the baseline control. (10 animals per group, P≤0.01 for all doses and time points).

The experiments in the ARPE-19 cell line indicated that 8-OH DPAT increased protection from oxidative stress and decreased the accumulation lipofuscin arising either form phagocytosis or autophagy, but would these effects have physiologic consequences in the retina? To examine possible in vivo protective actions, we employed the SOD2 knockdown model which exhibits an AMD-like phenotype [15]. Subretinal injection of the AAV-VMD2-*SOD2* Rz, which also contained the mCherry gene as a marker of genetic transduction routinely resulted in 60–80% transduction of the RPE which is in agreement with that previously reported [15] (Fig. 5). As a metric of neuroprotection we measured the full field scotopic ERG response at monthly intervals, beginning one month after subretinal injection of the viruses (Fig. 6a b). Subcutaneous administration of 8-OH DPAT improved the ERG response in *SOD2* knockdown eyes compared to knockdown eyes receiving vehicle control (Fig. 6a,b). In eyes injected with the control virus, AAV-VMD2-mCherry, we observed a modest decline (33%) in ERG amplitudes between the 1 month and 4 month time points. Treatment of the mice with 8-OH DPAT had no impact on the ERG response in these control-treated eyes. Injection of the AAV-VMD2-Rz432 (specific for *SOD2* mRNA) led to a 38% reduction in ERG amplitudes relative to the control treated eyes by one month post-injection. This decrease relative to control injection remained constant throughout the time course. Systemic treatment of the mice with 8-OH DPAT had a significant impact on the ERG response in eyes injected with the *SOD2* ribozyme. By one month after virus injection, a-wave amplitudes in mice treated with either the low-dose or the high dose of drug were elevated over 80% compared to saline treated mice (P<0.01) (Fig. 6a). By four months post injection, a-wave amplitude was increased over 100% in low-dose animals and over 130% in mice treated with the high dose of 8-OH DPAT (P<0.001). Results for b-wave amplitudes were not as dramatic, but by 4 months these were also elevated by over 70% in 8-OH DPAT mice at both high and low dosages (Fig. 6b).

**Figure 7:**
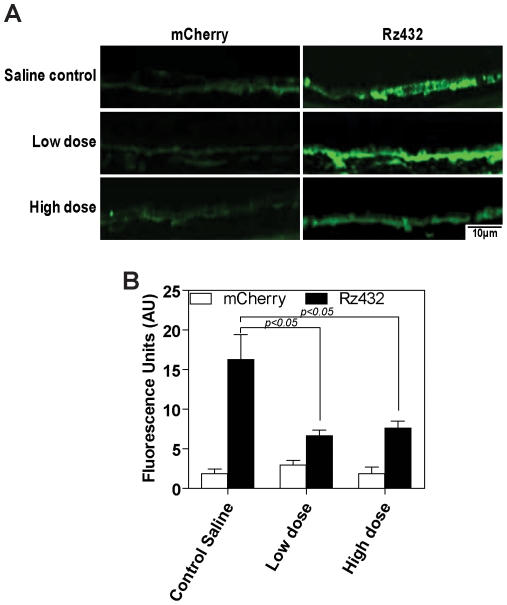
8-OH DPAT decreases oxidative stress in the RPE of SOD2 knockdown eyes of mice. Eyes received injection of AAV-*SOD2* ribozyme or AAV-mCherry and animals received subcutaneous 8-OH DPAT or saline control for up to 4 months. Sections were stained for 8-hydroxydeoxyguanosine (8OHdG) (green). **A** – Representative sections of 8OHdG expression in the RPE of control and SOD2 eyes receiving different concentrations of 8-OH DPAT. **B** - Graph shows quantitation of 8OHdG fluorescence in the RPE layer.

The simplest interpretation of the ERG results is that treatment of mice with 8-OH DPAT led to increased survival of photoreceptors in the face of RPE oxidative stress. Spectral domain OCT (SD-OCT) was used to measure the thickness of the outer nuclear layer, because this dimension reflects the survival of rod photoreceptor cells. We detected a small (7–14%) but statistically significant (p<0.01) difference in the ONL thickness in mice treated with the high dose of 8-OH DPAT (5 mg/kg) at 1–4 months past the time of *SOD2* mRNA knockdown (Fig. 6c). Low dose (0.5 mg/kg) also led to a statistically significant improvement in ONL dimensions at 3 and 4 months past injection (data not shown). *SOD2* knockdown resulted in a significant increase in the oxidative stress marker 8OHdG in both the neural retina and RPE (Fig. 7). However, this was significantly reduced in SOD2 knockdown eyes of animals receiving high dose 8-OH DPAT and reached levels of control eyes that received AAV-VMD2-mCherry (Fig. 7).

### 5-HT_1A_ agonist reduces lipofuscin accumulation in vivo

**Figure 8:**
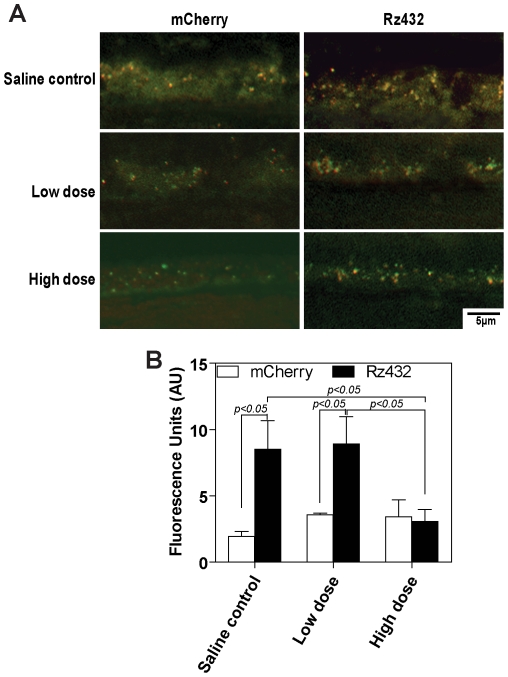
8-OH DPAT decreases RPE autofluorescence in SOD2 knockdown eyes of mice. Eyes received injection of AAV-*SOD2* ribozyme or AAV-mCherry and animals received subcutaneous 8-OH DPAT or saline control for up to 4 months. **A** - Confocal fluorescence microscopy of the RPE layers from AAV-mCherry and AAV-ribozyme treated eyes treated with saline, low dose or high dose of 8-OH DPAT. **B** - Treatment with 8-OH DPAT lowered autofluorescence in oxidatively stressed RPE in mice. Mice were injected with AAV-mCherry or AAV-Rz*SOD2*-mCherry and analyzed after 4 months of daily injections of saline (no treatment) low dose or high dose of 8-OH DPAT. * P<0.05, **P<0.01 (compared to AAV-RzSOD2 control).

In the *SOD2* knockdown model of dry AMD, loss of photoreceptors correlates with an increase in RPE autofluorescence [15]. To establish whether treatment with 8-OH DPAT reduced lipofuscin in ribozyme treated eyes, confocal microscroscopy was used to measure autofluorescence in the RPE layer 4 months after MnSOD knockdown (Fig. 8a). We noted a 60% reduction in autofluorescence, a measure of lipofuscin content of the RPE, in *SOD2* knockdown eyes treated with either the high or the low dose of 8-OH DPAT compared to eyes receiving vehicle alone (Fig. 8b).

## Discussion

Our results demonstrate the 5-HT_1A_ receptor agonist, 8-OH DPAT, is able to protect the retina from degeneration by reducing oxidative damage. We have shown that 8-OH DPAT can protect against oxidative stress by increasing antioxidant protection, reducing lipofuscin levels which, in turn, reduced the generation of ROS and prevented mitochondrial damage. Furthermore, 8-OH DPAT was able to confer neuroprotection in an animal model of AMD by reducing both oxidative stress and lipofuscin levels. These findings extend our previous observations in which we observed that 5-HT_1A_ receptor agonists were able to protect the retina from severe photo-oxidative stress, complement deposition and microglial activation [22], [23].

We used 8-OH DPAT in this study as it is a potent, and highly specific, 5-HT_1A_ receptor agonist [21] that has been extensively used to study neuroprotection in the brain [30], [31], [32], [33], [34]. The 5-HT_1A_ receptor is expressed in both the neural retina and the RPE [22], [23], [24] and we have previously reported that 8-OH DPAT compares well with other 5-HT_1A_ receptor agonists in providing retinal neuroprotection [22].

Both autophagy- and POS-derived lipofuscin accumulation was reduced by the 5-HT_1A_ receptor agonist in cultured ARPE19 cells. We, and others, have previously shown that autophagy-derived lipofuscin is a significant component of RPE lipofuscin [35], [36], [37] and that it is largely derived from the incomplete degradation of damaged organelles such as mitochondria which are prolific in the highly metabolically active RPE [9], [38], [39], [40].

Our data show that 8-OH DPAT is able to increase the levels of antioxidants such as MnSOD and glutathione which in turn reduce the generation of superoxide anions, diminish mitochondrial DNA damage and prevent the accumulation of oxidative endproducts such as MDA, 4HNE and nitrated protein. This increase in antioxidants improves mitochondrial function and reduces mitochondrial damage thereby reducing the autophagic turnover of these organelles. In addition, 5-HT has been shown to stimulate mTOR phosphorylation [41] and therefore 8-OH DPAT may be able to decrease autophagy induction. The 5-HT_1A_ receptor agonist was also able to induce a small, but significant reduction in POS-derived lipofuscin. The reason for this is unclear but could be due to either improved lysosomal function due to a reduction in autophagic load or reduced oxidative modification of ingested POS. Prior reduction in lipofuscin levels was maintained following removal of the 5-HT_1A_ receptor agonist from the culture system although, in the case of POS-derived lipofuscin, the rate of lipofuscin accumulation eventually recovered to that seen in untreated cultures. However, 8-OH DPAT did not lead to a major reduction in preformed lipofuscin indicating that the 5-HT_1A_ receptor agonist acts by inhibiting lipofuscinogenesis rather than reducing existing lipofuscin.

Activation of the 5-HT_1A_ receptor is neuroprotective in several in vitro [25], [42] and in vivo [30], [34], [43] model systems, but the mechanisms underlying these effects are incompletely understood. 5-HT1A receptor stimulation has been shown to lead to increased expression of several components of the antioxidant defense system, including SOD and catalase [44], [45], [46], anti-apoptotic proteins from the prosurvival members of the BCL family (e.g., Bcl-2 and Bcl-XL) [47], [48] and inhibitors of apoptosis proteins (e.g., Bax, XIAP) [47], [48], [49]. Similarly, we have shown that SOD2 is upregulated together with a decrease in the GSH/GSSG ratio in ARPE-19 cells by 5-HT_1A_ receptor activation. This, at least in part, explains the mechanism by which 8-OH DPAT reduced superoxide levels and protected against lipid peroxidation, nitrotyrosine levels and mitochondrial damage. The potential roles of upregulation of other endogenous defenses in RPE protection and lipofuscin inhibition remain to be explored.

Our *in vitro* observations were validated in our mouse model of atrophic AMD. We have previously shown that knockdown of SOD2 expression in the RPE leads to accumulation of lipofuscin and vacuolization and atrophy of the RPE culminating in death of overlying photoreceptor cells [15]. Systemic treatment with 8-OH DPAT sustained a- and b-wave ERG amplitudes in SOD2 knock down eyes compared to those not receiving 5-HT_1A_ receptor agonist. Furthermore, the loss in ONL thickness associated with SOD2 knock down was prevented in mice treated with 8 OH-DPAT. Taken together, these observations would suggest that systemic 5-HT_1A_ receptor agonist treatment preserves the survival of photoreceptor cells. This protection was associated with a decrease in the oxidative stress marker 8OHdG and reduced accumulation of lipofuscin in the RPE.

We conclude that 5-HT_1A_ receptor agonists offer a therapeutic option for retinal degenerations such as AMD, diabetic retinopathy or retinitis pigmentosa which involve oxidative stress and/or lipofuscin accumulation.

## Materials and Methods

### Cell Culture

The ARPE19 cell line was obtained from ATCC (CRL-2302). ARPE19 cells were grown to confluence as previously described [50], [51] and then maintained in basal medium (Ham's F10+2% fetal bovine serum (Invitrogen, Carlsbad, CA)) for 7 days prior to experimentation.

### *In vitro* lipofuscin formation

Lipofuscin formation in cultured RPE cells was achieved using our standard protocols [36], [52]. In brief, lipofuscin formation was induced in confluent, differentiated cultures of ARPE-19 by either a) maintaining cells for up to 28 days in basal medium (a measure of autophagic-derived lipofuscin accumulation) or b) incubating with isolated bovine photoreceptor outer segments (POS, 2×10^7^/ml) every two days for 14 days (a measure of phagocytic-derived lipofuscin accumulation).

### Lipofuscin isolation and phagocytosis by RPE

Lipofuscin granules were isolated and from 60–80 year old human donor eyes and purified by differential sucrose gradient centrifugation as previously described [53]. Lipofuscin granules were suspended in PBS and the concentration was determined by counting granules on a hemocytometer. Cultures were fed granules at a concentration of ∼300 granules/cell and maintained for 7 days prior to experimentation.

### 5HT_1A_ agonist treatment

To assess the ability of the 5-HT_1A_ receptor agonist, 8-OH DPAT (Sigma, St. Louis, MO, USA), to reduce lipofuscin formation in cultured RPE cells, this compound was added to the culture medium every 48 hours at concentrations ranging from 0.1 to 20 µM. All experiments were undertaken in basal medium (Ham's F10+2% FBS), and cells receiving vehicle alone (PBS) acted as negative controls. To determine if the effect of 8-OH DPAT was sustained following discontinuation of 5-HT_1A_ receptor agonist treatment, 8-OH DPAT treatment was discontinued after 28 days and the cells maintained in basal medium or fed POS for a further 28 days. To assess the ability of 8-OH DPAT to remove existing lipofuscin, autophagy-derived lipofuscin and phagocytic-derived lipofuscin were allowed to accumulate as described above and then 8-OH DPAT was added every second day for up to 28 days. To confirm that 8-OH DPAT was acting via the 5-HT_1A_ receptor agonist we included the 5-HT_1A_ receptor antagonist S(-)-UH-301 at 5 µM in some experiments. To determine the effect of timing of 8-OH DPAT treatment on oxidative stress markers, RPE cultures were either pre-treated with 8-OH DPAT (at 1 or 10 µM) for 3 or 24 hours prior to exposure to 200 µM H_2_O_2_ for 1 hour or treated with the 5HT_1A_ agonist for 3 or 24 hours post-exposure to H_2_O_2_. Cells not exposed to oxidative stressor served as a negative control, and cells exposed to oxidative stressor but not 8-OH DPAT served as a positive control. Cells exposed to 8-OH DPAT only acted as an additional control.

### Analysis of lipofuscin accumulation in cultured RPE cells

Lipofuscin levels in cultured cells were assessed by fluorescence microscopy and quantified by both FACS and image analysis at time 0 and weekly thereafter as previously described [36], [52]. For FACS analysis, cells were resuspended in phosphate-buffered saline (PBS) at 1×10^5^ cells/ml. The mean autofluorescence per 10,000 RPE cells was determined using a fluorophotometric flow cytometer, excitation at 488 nm and emission at 600 nm. Fluorescence microscopy images of cultured cells were obtained using an inverted microscope equipped with 450 nm to 490 nm excitation and 520-nm barrier filters. Fluorescence intensity of the intracellular, autofluorescent granules was assessed by ImageJ program available at: http://rsbweb.nih.gov/.

### Superoxide anion assay

Superoxide generation was measured using FACS analysis following staining with MitoSOX (Molecular Probes, Invitrogen, Carlsbad, CA ) and results are expressed as the mean fluorescence intensity.

### Cytochrome Oxidase, 4HNE/MDA and nitrosylation assays

Cytochome *c* oxidase levels were measured using a Complex IV ELISA assay (MitoSciences, Eugene, Oregon, USA). The level of the lipid peroxides 4 hydroxynonenal (4HNE) and malonaldehyde (MDA) were determined using a commercially available kit from OxisResearch (Burlinghame, CA, USA). The LPO-586 method is based on the reaction of the chromogenic reagent N-methyl-2-phenyindole with MDA and 4HNE. Protein nitration was assessed by Western blotting using the polyclonal α-nitrotyrosine antibody from Millipore (Billerica, MA, USA) and normalized against β-actin.

### MnSOD and Glutathione assays

MnSOD levels were determined by Western blot analysis and the ratio of reduced glutathione (GSH) to the oxidized form of glutathione (GSSG) was measured by ELISA using a kit from OxisResearch (Burlinghame, CA, USA).

### Mitochondrial DNA damage assay

Mitochondrial DNA (mtDNA) damage was analyzed as previously described using the long chain PCR reaction [51]. In brief, genomic DNA was extracted and qPCR was performed to determine the average oxidative lesion frequency. The PCR conditions used in this study were based on published data using previously reported sequences for mtDNA primers [51], [54]. qPCR was carried out with all reactions being a total of 100 µl containing 15 ng of total genomic DNA, 1 unit of XL r*Tth* polymerase, 3.3 XL PCR buffer II (containing potassium acetate, glycerol, and DMSO), and final concentrations of 200 µM dNTPs, 1.2 mM Mg(AOC)_2_ and 0.1 µM primers. The gene fragments were amplified and resolved on a 1% agarose gel. The intensity of the PCR product bands was quantified with Scion Image analysis software (Scion Corp., version Beta 4.0.2).

### Animals and injections

All animal studies were performed under a protocol approved by the Institutional Animal Care and Use Committee at the University of Florida , (IACUC Study #201005197), and in accordance with the NIH Guide for Care and Use of Laboratory Animals and ARVO Statement for the Use of Animals in Ophthalmic and Vision Research. We used the previously described *SOD2* knockdown model that exhibits an AMD-like phenotype including electroretinogram changes, basal deposits, elevated lipofuscin accumulation, thickening of Bruch's membrane and patchy regions of RPE atrophy overlying photoreceptor degeneration [15]. The right eyes of C57Bl/6 mice were injected with Adeno-associated virus (AAV) expressing a ribozyme that cleaves the mRNA for mitochondrial superoxide dismutase (MnSOD encoded by the *SOD2* gene). Expression of the ribozyme was restricted to the RPE using the *VMD2* (bestrophin) promoter and by subretinal injection with AAV serotype 1. This virus also expressed the marker gene, mCherry, to determine the extent and location of virus infection. The left eyes were injected with virus expressing only mCherry. All mice were housed in a 12 h∶12 h light/dark cycle under specific pathogen-free (SPF) conditions. Subretinal AAV injections were performed as described by Timmers et al. [55]. C57Bl/6 mice were injected at postnatal day 15 (P15). For this purpose, mice were anesthetized by ketamine/xylazine injection. Pupils were dilated with 1 drop of 1% atropine sulfate and 2.5% phenylephrine. Right eyes were injected in the superior hemisphere with 1 µl AAV-*VMD2 Rz432* mCherry (2×10^9^ vector genomes). Left eyes were injected with AAV-VMD2-mCherry at the same titer.

### Effect of 8-OH DPAT in the SOD2 knockdown model for AMD

Mice were randomly assigned to one of three groups and received daily subcutaneous injections of either sterile saline, 0.5 mg/kg body weight 8-OH-DPAT in sterile saline (low dose), or 5.0 mg/kg 8-OH-DPAT (high dose). At monthly intervals mice were evaluated by digital fundus imaging, full-field scotopic (dark adapted) electroretinography (ERG) and spectral domain optical coherence tomography (SD-OCT). At four months, mice were euthanized and eyes were prepared for cryosectioning. Confocal microscroscopy was used to measure autofluorescence in the RPE layer of untreated eyes and virus-treated eyes with or without 8OH-DPAT. Excitation frequency was 387 nm and emission frequency was 440–684 nm and fluorescence intensity was assessed using ImageJ. Sections were also used to assess damage to retinal structure by light microscopy and immunostaining for 8-Oxo-2′-deoxyguanosine (8OHdG) (Abcam, Cambridge, MA, USA) as a measurement of oxidative stress using previously described methodology [56].

### Statistics

All experiments were repeated at least three times. FACS, autofluorescence, antioxidant, ERG and SD-OCT data were compared using a Student's *t* test plus ANOVA with Bonferroni correction for multiple comparisons. The Mann-Whitney test was used to determine statistical significance in the Western blotting analysis, qPCR. Results are expressed as mean±SEM. *p*<0.05 is considered statistically significant.
